# Indirect comparison of azacitidine and decitabine for the therapy of elderly patients with acute myeloid leukemia: a systematic review and network meta-analysis

**DOI:** 10.1186/s40164-020-00160-8

**Published:** 2020-03-16

**Authors:** Bingbing Wen, Weiwen You, Sitian Yang, Xin Du

**Affiliations:** 1 Department of Internal Medicine, Shenzhen Second People’s Hospital, The First Affiliated Hospital of Shenzhen University, Shenzhen, 518000 China; 2 Department of Hematology, Shenzhen Second People’s Hospital, The First Affiliated Hospital of Shenzhen University, 3002 Sungang West Road, Futian District, Shenzhen, 518000 China

**Keywords:** Azacitidine, Acute myeloid leukemia, Elderly patients, Decitabine, Network meta-analysis

## Abstract

**Background:**

The DNA hypomethylating agents (HMAs) decitabine and azacitidine have been widely used in the management of elderly patients with acute myeloid leukemia (AML). However, no direct clinical trials have been carried out to compare the two agents. A systematic review and network meta-analysis were performed to indirectly compare the efficacy and safety of decitabine and azacitidine in elderly AML patients.

**Methods:**

We systematically searched PubMed, Medline, Web of Science, Embase and Cochrane Library through May 14, 2019. Randomized controlled trials on elderly AML patients comparing the efficacy and safety between decitabine and azacitidine, or comparing one of HMAs to standard supportive care or placebo were selected. The major outcomes of interest were performed with methods of adjusted indirect comparison and the fixed effect model.

**Results:**

Only three RCTs including a total number of 1086 patients were identified. Direct comparisons showed that azacitidine significantly reduced mortality (RR = 0.90, 95% CI 0.83–0.97) while decitabine was not significantly associated with lower mortality (RR = 0.97, 95% CI 0.92–1.02) compared to the conventional care regimen (CCR). In addition, for the indirect method, azacitidine significantly reduced mortality compared to decitabine (RR = 0.83 95% CI 0.77–0.90) and was more likely to improve complete response (CR) (RR = 1.66, 95% CI 1.17–2.35, low-certainty evidence). No statistical significance was found for the other studied outcomes.

**Conclusions:**

Compared to CCR, decitabine and azacitidine can promote studied outcomes in elderly AML patients. Indirect evidence with low certainty was used to compare these two agents. The superiority of either agent cannot be confirmed, and head-to-head clinical trials are still required.

## Background

Acute myeloid leukemia (AML), characterized by the expansion of clonal myeloid cells in the bone marrow and peripheral blood, is a heterogeneous haematologic malignancy with clinical manifestations of anaemia, haemorrhage and infection [1]. The majority of AML cases are elderly patients with a median diagnosed age of 67 years presented by the SEER Cancer Statistics Review [2]. However, poor prognosis and limited treatments for elderly patients account for the largest number of annual deaths especially for those with comorbidities and poor performance status [3]. The annual incidence rates of AML since from 2010 have been are consistently higher than 4.2 per 100,000 per year [4–8]. The 2- and 5-year overall survival (OS) rates of elderly AML patients are approximately 10% and 2%, respectively [9–11]. Expert bodies, such as the National Comprehensive Cancer Network (NCCN) and European Leukemia Net (ELN), suggest recommended regimens for older and less fit AML patients, including azacitidine and decitabine as one of the options [12, 13].

Hypomethylating agents (HMAs), including decitabine and azacitidine, are pyrimidine nucleoside analogues of cytidine and have been approved to treat the AML patients by the Food and Drug Administration (FDA). The use of HMAs is still a common method for those AML patients who are unfit to receive intensive treatment or haematopoietic stem cell transplant (HSCT) [14]. Superior responses have been proven both with azacitidine and decitabine compared to supportive care alone [15–17]. However, direct comparison of these two agents has not been performed in a randomized trial, and the selection of treatments of two agents is still a dilemma for patients and physicians. Therefore, the objective of this study was to compare the efficacy and safety of azacitidine and decitabine in elderly AML patients by performing a systematic review and network meta-analysis.

## Methods

This systematic review and meta‐analysis follow the guidelines of Preferred Reporting Items for Systematic Reviews and Meta-Analyses (PRISMA) [18].

### Search strategies

We systematically searched all studies published electronically in Medline, PubMed, Web of Science, Cochrane Library and Embase through May 14, 2019, without time or language restrictions. The keywords we used in the research were: “acute myeloid leukemia,” “azacitidine,” “decitabine,” “elderly patients,” and “randomized controlled trial.” Two study researchers designed and performed the search strategy (Additional file 1: Table S4).

### Eligibility criteria

Only randomized controlled trials (RCTs) were selected in our systematic review and network-meta analysis. A trial that investigated elderly patients diagnosed with acute myeloid leukemiaand included treatment with azacitidine or decitabine, and compared the two drugs against each other, or compared them to standard supportive care, or placebo was selected. In addition, at least one of the relevant outcomes should be reported in the trial including: mortality, complete and partial responses, and haematologic improvement. We excluded review articles and nonrandomized control trials.

### Study selection

Two review authors screened all the titles and abstracts of trials independently and indicated the eligibility based on inclusion and exclusion criteria. Full text articles and their relevant references were selected for further assessment. Disagreements were settled by discussion of the two review authors and involved a third independent reviewer if necessary.

### Data extraction

Two reviewer authors (BW and WY) extracted data independently from the included studies including study characteristics, participant information of participants, intervention, and interesting outcomes. Disagreements were settled by discussion of the two reviewers. All data were recorded in Microsoft Excel (2016).

### Methodologic quality and risk of bias

Based on the Cochrane Collaboration’s tool, the methodological quality of included trials and risk of bias were evaluated by two review authors, which included seven domains: allocation concealment, random sequence generation, incomplete outcome data, selective outcome reporting, blinding of participants and personnel, blinding of outcome assessment and other bias. The risk of bias was rated as high, unclear, or low. Two review authors (BW, WY) evaluated the risk of bias in each trial independently and disagreements were settled by discussion with co-authors.

### Quality of evidence

The quality of evidence, also called certainty in evidence, was evaluated with the GRADE (Grading of Recommendations, Assessment, Development and Evaluation) working group. There were four levels of quality of evidence: very low, low, moderate, and high. Randomized control trials were regarded as high quality but could be downgraded due to indirectness, inconsistency, imprecision, risk of bias and publication bias [19].

### Statistical analysis

All the network meta-analyses (NMAs) were performed by using STATA 14.0 software (Stata Corporation, Texas) and Review Manager 5.3 software (Cochrane Collaboration, Oxford, UK). The binomial distribution was used to calculate and express with relative risks (RRs) and a 95% confidence intervals (95% CIs). Because the number of trials was less than 3 and the potential heterogeneity was set among studies, Mantel and Haenszel (M-H) fixed effects models were carried out, and I^2^ was used to detect the heterogeneity [20]. *I*^2^ statistics greater than 50% represent substantial statistical heterogeneity. The graph and summary of risk of bias were created to assess the bias within studies.

The adjusted indirect comparisons were performed with Relative Risk (RRs) and 95% Confidence Interval (CIs) to assess indirect comparisons of two agents [21]. We used the surface under the cumulative ranking (SUCRA) probabilities to rank the treatments for an outcome [22, 23]. For the survival outcome of elderly patients with AML, the largest SUCRA scores might indicate the best intervention.

## Results

### Included studies

**Fig. 1:**
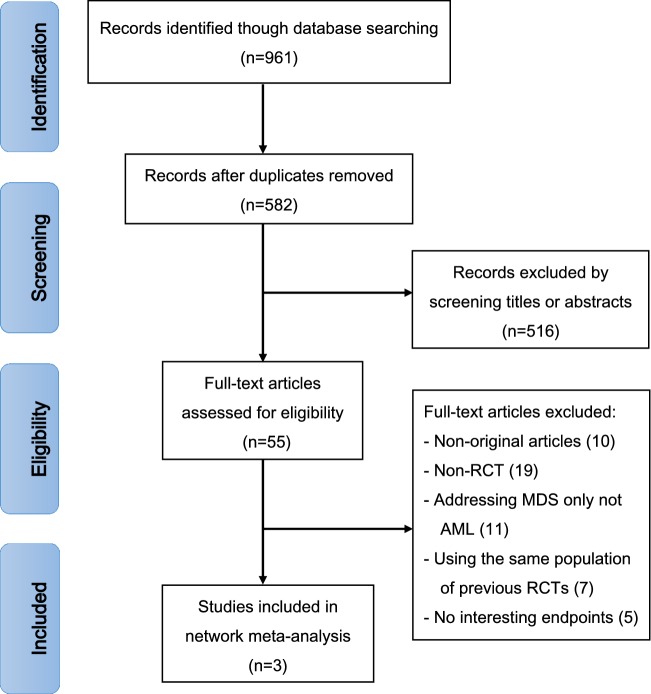
Flowchart presenting the steps of the literature search and selection

**Fig. 2:**
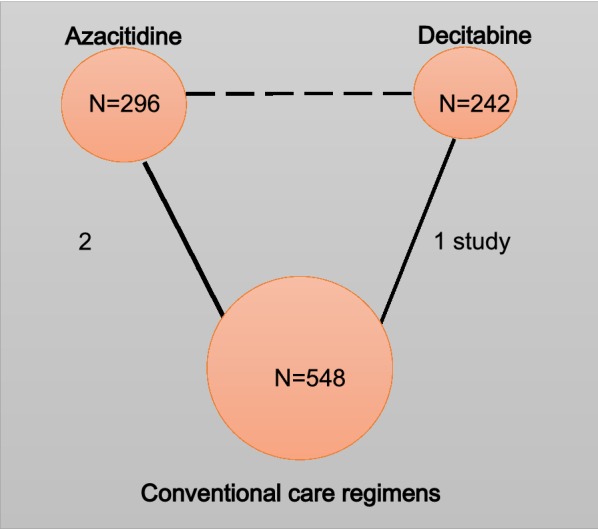
Network plots of the AML network. Nodes are weighted according to the total number of patients in the included studies. The dashed line represents indirect evidence. Solid lines represent direct evidence

A total of 961 records were obtained with the electronic search strategy. After removing duplicates, 582 articles were screened by title and abstract. A total of 516 trials were excluded due to ineligibility and 55 citations were included for full-text analysis. Finally, three trials were eligible for extraction for this network meta-analysis (Fig. 1). The AML network plot was shown in Fig. 2.

### Study characteristics

The three RCTs involved a total number of 1086 patients with an age range of 64–91 years old. Two RCTs compared of azacitidine (75 mg/m^2^/day, SC × 7 days) and the conventional care regimens (CCR), including low-dose cytarabine (LDAC) or best supportive care (BSC) or intensive chemotherapy (IC), and included 601 patients (296 azacitidine and 305 CCR; age average 74; range 64–91 years old). The other RCT compared decitabine (20 mg/m^2^, IV, QD × 5 days/4 weeks) to the CCR including supportive care or cytarabine and included 485 patients (242 decitabine and 243 CCR; age average 73; range 64–91 years old) (Table 1).

**Table 1:** Characteristics of included studies

Author, year	Intervention (dose, schedule)	Comparison (description)	Patients (N)	Age (years)	Male (%)	Cytogenetic risk group, N (%)	ECOG score	Bone marrow blasts, median (range)	Follow-up (months)
Azacitidine									
Fenaux et al. [15]	Azacitidine: subcutaneously 75 mg/m^2^/day for 7 days Q28 days for at least 6 cycles	CCR (BSC, LDAC 20 mg/m^2^/day for 14 days Q28 days for at least 6 cycles, IC)	Intervention: 55 Comparison: 58	Intervention: 73 (64–89) Comparison: 73 (64–91)	Intervention: 62.1 Comparison: 56.6	Intermediate: 81 (71.7%) Normal: 52 (46.0%) Poor risk: 27 (23.9%) Missing: 5 (4.4%)	0–1: 107 (94.7%) 2: 4 (3.5%) Missing: 2 (1.8%)	Intervention: 23.0 (20.0–34.0) Comparison: 23.1 (13.0–68.9)	40
Dombret et al. [16]	Azacitidine: subcutaneously 75 mg/m^2^/day for 7 days Q28 days for at least 6 cycles	CCR (BSC, LDAC 20 mg/m^2^/day for 14 days Q28 days for at least 6 cycles, IC)	Intervention: 241 Comparison: 247	Intervention: 75 (64–91) Comparison: 75 (65–89)	Intervention: 57.7 Comparison: 60.3	Intermediate: 306 (63.1%) Normal: – Poor risk: 174 (35.8%) Missing: –	0-1: 375(76.8%) 2: 113(23.2%) missing:–	Intervention: 70.0 (2.0–100.0) Comparison: 72.0 (2.0–100.0) > 50%: 366 (75.0%)	40
Decitabine									
Kantarjian et al. [17]	Decitabine: intravenously 20 mg/m^2^ QD for 5 days, every 4 weeks	TC (supportive care,or cytarabine 20 mg/m^2^ QD for 10 days, every 4 weeks)	Intervention: 242 Comparison: 243	Intervention: 73 (64–89) Comparison: 73 (64–91)	Intervention: 62.1 Comparison: 56.6	Intermediate: 306 (63.1%) Normal: – Poor risk: 174 (35.8%) Missing: –	0–1: 367 (75.7%) 2: 118 (24.3%) Missing: –	20–30%: 123 (25.2%) > 30–50%: 141 (29.3%) > 50%: 206 (42.7%)	36

*CCR* conventional care regimens, *BSC* best supportive care, *LDAC* low-dose ara-c, *IC* induction chemotherapy, *TC* treatment choice, *N* total number of patients, *ECOG* Eastern Cooperative Oncology Group performance status

### Outcomes

**Fig. 3:**
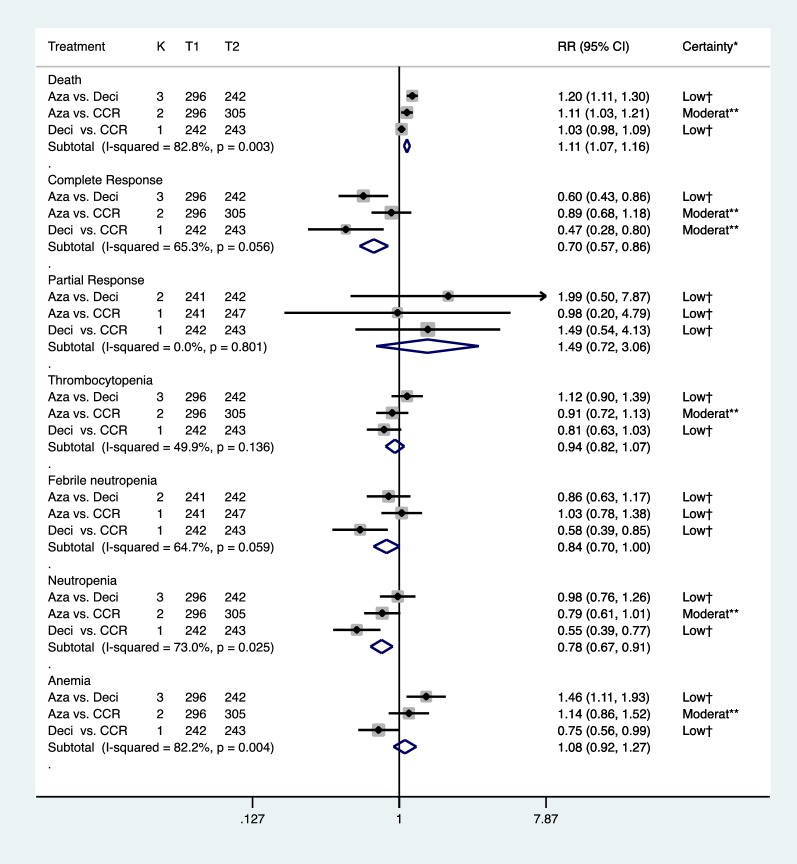
Forest plot represents the direct and indirect comparison. RR, relative risks; 95%Cis, 95% confidence intervals; CCR, conventional care regimens; K, total number of RCTs; T, total number of patients. Certainty*: certainty in evidence. ^†^Rated down for imprecision and methodological limitation. **Rated down for methodological limitation (unclear risk of bias).

**Fig. 4:**
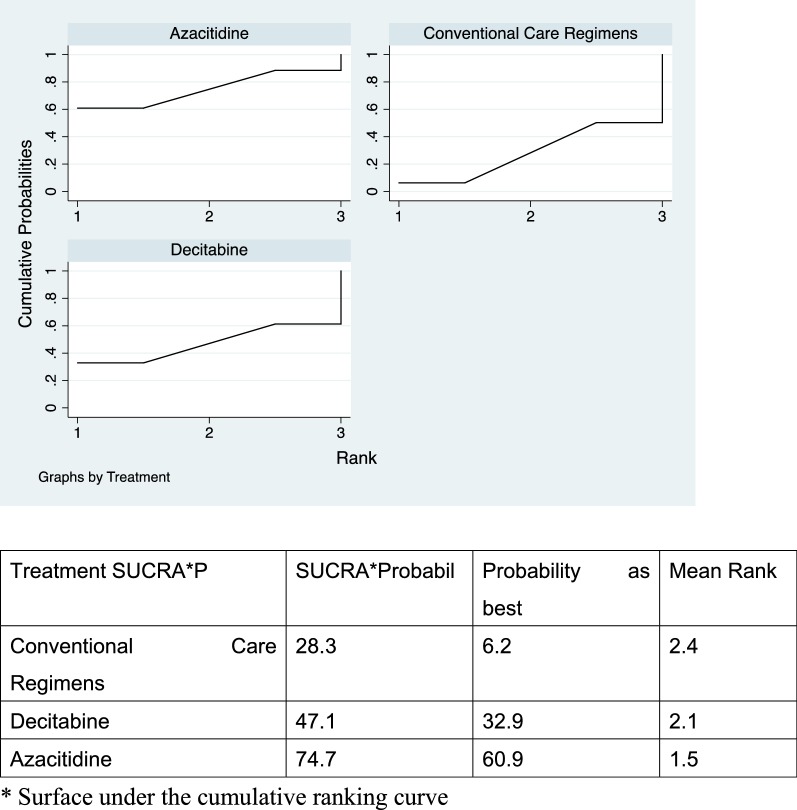
The surface under the cumulative ranking curves for survival outcome

Direct comparisons showed that azacitidine significantly reduced mortality (RR = 0.90, 95% CI 0.83–0.98, p < 0.001, I^2^ = 94.0%) (Additional file 1: Figures S1 and S2), while decitabine did not show improvement in mortality rates compared to CCR (RR = 0.97, 95% CI 0.92–1.02) (Additional file 1: Figures S3 and S4). Higher complete responses were reported in both groups as compared to CCR. In addition, indirect head-to-head comparisons showed that azacitidine significantly reduced the mortality rate (RR = 0.83 95% CI 0.77–0.90, I^2^ = 82.8%) and anemia (RR = 0.68, 95% CI 0.52–0.90, I^2^ = 82.2%). Patients in the azacitidine group were more likely to achieve complete response (CR) compared to decitabine (RR = 1.66, 95% CI 1.17–2.35, I^2^ = 65.3%, low certainty) (Fig. 3). There was no statistically significant difference found in other study outcomes including partial response rate, neutropenia and thrombocytopenia. Similarly, azacitidine showed improved overall survival by SUCRA analysis compared to decitabine (74.7% vs. 47.1%) (Fig. 4).

### Methodologic quality and risk of bias

**Fig. 5:**
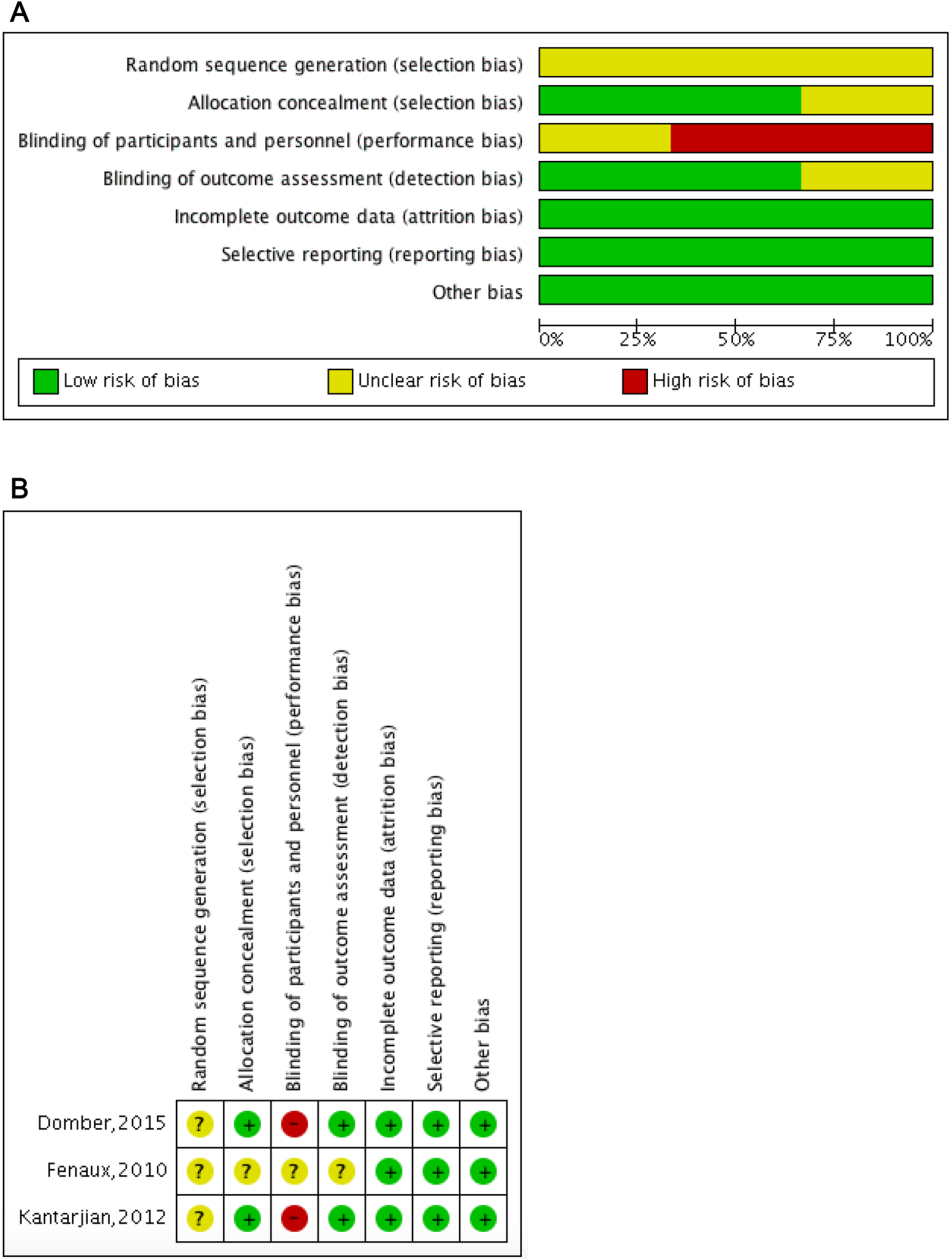
**a** Risk of bias graph. **b** Risk of bias summary

The risk of bias among studies was categorized as low, unclear, or high to its ranking. All the trials were unclear as for random sequence generation. Allocation concealment and blinding of outcome assessment were both achieved in the two trials, whereas blinding of participants and personnel was not conducted in the same trials due to the open label design. In addition, selective reporting and incomplete outcome data were low risk in all trials. The graph and summary of the risk of bias are shown in Fig. 5.

### Certainty in evidence

The unclear risk of bias was shown in all outcomes of the included studies and publication bias could not be explored because of the small number of the trials for direct comparisons, which results in downgrading the certainty. Therefore, low or moderate of the certainty in evidence was found for various outcomes to support the efficacy and safety of azacitidine or decitabine compared to conventional care regimens (Fig. 3 and certainty in the evidence Additional file 1: Tables S1–S3). Similarly, with imprecision and unclear risk of bias, low certainty for various outcomes was performed in head-to-head comparison. The consistency of the network could not be evaluated because there were no closed loops.

## Discussion

In our systematic review and network meta-analysis, three RCTs including 1086 patients were included. Treating patients with azacitidine or decitabine provided improved outcomes in terms of mortality, overall response rate, and improvement in haematological parameters. Indirect head-to-head comparison, with low certainty in evidence, showed that azacitidine was superior to decitabine in terms of the overall survival.

HMAs are still common methods for elderly AML patients who are unfit for intensive therapy or HSCT [24]. NCCN recommendations list treatment with azacitidine and decitabine for elderly patients with newly diagnosed AML as an option [13]. Tapan et al. [25] showed that decitabine could enhance outcomes in elderly AML patients (CR/CRi rate and median of OS were 27% and 8.6 months, respectively). Furthermore, a randomized study conducted by Yanis Boumber et al. suggested decitabine rather than conventional care regimens for maintenance treatment in AML patients with complete remission, and the OS rates in the decitabine and CCR groups were 45% and 36%, respectively) [26]. A recent study showed no difference in safety and efficacy between the 5-day decitabine treatment and the 10-day decitabine treatment in elderly patients with AML [27]. Similarly, compared to CCR, Seymour et al. [28] in 2017 suggested that azacitidine could enhance clinical outcomes in elderly AML patients with myelodysplasia-related changes. In this study, direct comparison showed that the use of either azacitidine or decitabine resulted in lower mortality and higher complete responses compared to CCR, which are consistent with the results of the above trials.

However, no randomized trial has been ever conducted directly to compare azacitidine and decitabine in elderly AML patients. Mehra et al. [29] recently conducted an analysis of comparable survival outcomes and showed that the median OS of decitabine or azacitidine used for frontline treatment in elderly AML patients who were unfit for intensive chemotherapy was comparable. In this study, low certainty of the evidence was found when comparing azacitidine and decitabine. The different baseline characteristics of the studies may have influenced the different results of the trials. Many oncology providers still sometimes face dilemmas when deciding between different hypomethylating agents. Furthermore, factors including preferences of patients, adverse effects, and cost can be taken under consideration in the final therapy of two agents.

## Limitations

There were some limitations in this study. The consistency of the network could not be evaluated because there were no closed loops. Heterogeneity and publication bias could not be obtained because of the small number of trials investigating each agent. In addition, direct and indirect head-to-head comparisons were performed with low or moderate of the certainty of the evidence. Subgroup analysis could not be assessed due to the paucity of data.

## Conclusions

Compared to CCR, azacitidine or decitabine yields both better outcomes, including mortality, overall response, and improvement of haematological parameters. For indirect head-to-head comparisons, low certainty of evidence was found when comparing azacitidine and decitabine. The superiority of either agent cannot be confirmed in this study and head-to-head clinical trials are still required to provide more information about the efficacy and safety of the two agents. In addition, other factors including adverse effects, patient preferences and cost, are also important and should be taken into consideration in the final choice between the two agents.

## Supplementary information

**Additional file 1: Figure S1.** Azacitidine vs. conventional care regimens (direct evidence-RR). **Figure S2.** Azacitidine vs. conventional care regimens (direct evidence-OR). **Figure S3.** Decitabine vs. conventional care regimens (direct evidence-RR). **Figure S4.** Decitabine vs. conventional care regimens (direct evidence-OR). **Table S1.** Azacitidine compared to CCR for elderly patients diagnosed with AML. **Table S2.** Decitabine compared to CCR for elderly patients diagnosed with AML. **Table S3.** Azacitidine compared to decitabine for elderly patients diagnosed with AML. **Table S4.** Search strategies.

## Data Availability

Not applicable.

## Abbreviations

HSCT: Hematopoietic stem cell transplantation
BSC: Best supportive care
CCR: Conventional care regimens
HMA: Hypomethylating agent
AML: Acute myeloid leukemia
RCT: Randomized controlled trial
