# Supplementary material for: Indirect comparison of azacitidine and decitabine for the therapy of elderly patients with acute myeloid leukemia: a systematic review and network meta-analysis
Source: Exp Hematol Oncol. 2020 Mar 16;9:3. doi: 10.1186/s40164-020-00160-8 (PMC7075015; doi:10.1186/s40164-020-00160-8)
Supplement: Supplementary file 1 — Additional file 1: Figure S1. Azacitidine vs. conventional care regimens (direct evidence-RR). Figure S2. Azacitidine vs. conventional care regimens (direct evidence-OR). Figure S3. Decitabine vs. conventional care regimens (direct evidence-RR). Figure S4. Decitabine vs. conventional care regimens (direct evidence-OR). Table S1. Azacitidine compared to CCR for elderly patients diagnosed with AML. Table S2. Decitabine compared to CCR for elderly patients diagnosed with AML. Table S3. Azacitidine compared to decitabine for elderly patients diagnosed with AML. Table S4. Search strategies. [file 40164_2020_160_MOESM1_ESM.docx]

**Indirect comparison of azacitidine and decitabine for the therapy of elderly patients with acute myeloid leukemia: a systematic review and network meta-analysis**

**(Appendix)**

Table of Contents

*Supplemental Figures* 2

*Figure S1: Azacitidine vs. Conventional Care Regimens (Direct Evidence-RR)* 2

*Figure S2: Azacitidine vs. Conventional Care Regimens (Direct Evidence-OR)* 3

*Figure S3: Decitabine vs. Conventional Care Regimens (Direct Evidence-RR)* 4

*Figure S4: Decitabine vs. Conventional Care Regimens (Direct Evidence-OR)* 5

*Certainty in the evidence tables (Evidence profiles)* 6

*Table S1:Azacitidine compared to CCR for adults diagnosed with AML* 6

*Table S2:Decitabine compared to CCR for adults diagnosed with AML* 7

*Table S3: Azacitidine compared to decitabine for adults diagnosed with AML* 8

*Search Strategies:* 9

**Supplemental Figures**

**Figure S1: Azacitidine vs. Conventional Care Regimens (Direct Evidence-RR)**

**

Forest plot represents the direct comparison. RR, relative risks; 95%Cis, 95% confidence intervals; CCR, conventional care regimens; n, total number of events; T, total number of patients.

**Figure S2: Azacitidine vs. Conventional Care Regimens (Direct Evidence-OR)**

Forest plot represents the direct comparison. OR, odds ratio; 95%Cis, 95% confidence intervals; CCR, conventional care regimens; n, total number of events; T, total number of patients.

**Figure S3: Decitabine vs. Conventional Care Regimens (Direct Evidence-RR)**

Forest plot represents the direct comparison. RR, relative risks; 95%Cis, 95% confidence intervals; CCR, conventional care regimens; n, total number of events; T, total number of patients.

**Figure S4: Decitabine vs. Conventional Care Regimens (Direct Evidence-OR)**

Forest plot represents the direct comparison. RR, relative risks; 95%Cis, 95% confidence intervals; CCR, conventional care regimens; n, total number of events; T, total number of patients.

**Certainty in the evidence tables (Evidence profiles)**

**Table S1: Azacitidine compared to CCR for elderly patients diagnosed with AML**

|  | **No. of patients** | | **Certainty assessment** | | | | | | **Effect** | **Certainty** | **Importance** |
| --- | --- | --- | --- | --- | --- | --- | --- | --- | --- | --- | --- |
| **No of studies** | **azacitidine** | **CCR** | **Study**  **design** | **Risk of bias** | **Inconsistency** | **Indirectness** | **Imprecision** | **Other**  **considerations** | **Relative**  **(95% CI)** |  |  |
| Death (follow up: median 40 months) | | | | | | | | | | | |
| 2 | 223/296  (75.3%) | 256/305  (84.0%) | RCT | serious* | not serious | not serious | not serious | none | RR 0.90  (0.83-0.97) | ⨁⨁⨁◯  Moderat | CRITICAL |
| Complete response (follow up: median 40 months) | | | | | | | | | | | |
| 2 | 77/296  (26.0%) | 71/305  (23.3%) | RCT | serious* | not serious | not serious | not serious | none | RR 1.12  (0.84-1.48) | ⨁⨁⨁◯  Moderat | CRITICAL |
| Partial response (follow up: median 40 months) | | | | | | | | | | | |
| 1 | 3/241  (1.2%) | 3/247  (1.2%) | RCT | serious* | not serious | not serious | serious** | none | RR 1.02  (0.21-5.03) | ⨁⨁◯◯  Low | CRITICAL |
| Anemia (follow up: median 40 months) | | | | | | | | | | | |
| 2 | 67/296  (22.6%) | 79/305  (25.9%) | RCT | serious* | not serious | not serious | not serious | none | RR 0.87  (0.66-1.16) | ⨁⨁⨁◯  Moderat | CRITICAL |
| Febrile Neutropenia (follow up: median 40 months) | | | | | | | | | | | |
| 1 | 66/241  (27.3%) | 70/247  (28.3%) | RCT | serious* | not serious | not serious | serious** | none | RR 0.97  (0.73-1.29) | ⨁⨁◯◯  Low | CRITICAL |
| Neutropenia (follow up: median 40 months) | | | | | | | | | | | |
| 2 | 95/296  (32.1%) | 77/305  (25.2%) | RCT | serious* | not serious | not serious | not serious | none | RR 1.27  (0.99-1.64) | ⨁⨁⨁◯  Moderat | CRITICAL |
| Thrombocytopenia (follow up: median 40 months) | | | | | | | | | | | |
| 2 | 104/296  (35.1%) | 97/305  (31.8%) | RCT | serious* | not serious | not serious | not serious | none | RR 1. 10  (0.88-1.38) | ⨁⨁⨁◯  Moderat | CRITICAL |

*Confidence interval; RR: Risk ratio; CCR Conventional Care Regimens*

**Inadequate allocation concealment and blinding in some trials*

*** Confidence intervals that includes appreciable benefits and harms*

**Table S2: Decitabine compared to CCR for elderly patients diagnosed with AML**

|  | **No. of patients** | | **Certainty assessment** | | | | | | **Effect** | **Certainty** | **Importance** |
| --- | --- | --- | --- | --- | --- | --- | --- | --- | --- | --- | --- |
| **No of studies** | **decitabine** | **CCR** | **Study**  **design** | **Risk of bias** | **Inconsistency** | **Indirectness** | **Imprecision** | **Other**  **considerations** | **Relative**  **(95% CI)** |  |  |
| Death (follow up: median 36 months) | | | | | | | | | | | |
| 1 | 219/242  (90.5%) | 227/243  (93.4%) | RCT | serious* | not serious | not serious | serious** | none | RR 0.97  (0.92-1.02) | ⨁⨁◯◯  Low | CRITICAL |
| Complete response (follow up: median 36 months) | | | | | | | | | | | |
| 1 | 38/242  (15.7%) | 18/243  (7.4%) | RCT | serious* | not serious | not serious | serious** | none | RR 2.12  (1.25-3.61) | ⨁⨁⨁◯  Moderat | CRITICAL |
| Partial response (follow up: median 36 months) | | | | | | | | | | | |
| 1 | 6/242  (2.4%) | 9/243  (3.7%) | RCT | serious* | not serious | not serious | serious** | none | RR 0.67  (0.24-1.85) | ⨁⨁◯◯  Low | CRITICAL |
| Anemia (follow up: median 36 months) | | | | | | | | | | | |
| 1 | 80/242  (33.1%) | 60/243  (24.6%) | RCT | serious* | not serious | not serious | serious** | none | RR 1.34  (1.01-1.78) | ⨁⨁◯◯  Low | CRITICAL |
| Febrile Neutropenia (follow up: median 36 months) | | | | | | | | | | | |
| 1 | 57/242  (23.6%) | 33/243  (13.6%) | RCT | serious* | not serious | not serious | serious** | none | RR 1.73  (1.17-2.56) | ⨁⨁◯◯  Low | CRITICAL |
| Neutropenia (follow up: median 36 months) | | | | | | | | | | | |
| 1 | 76/242  (31.4%) | 42/243  (17.3%) | RCT | serious* | not serious | not serious | serious** | none | RR 1.82  (1.30-2.56) | ⨁⨁◯◯  Low | CRITICAL |
| Thrombocytopenia (follow up: median 36 months) | | | | | | | | | | | |
| 1 | 95/242  (39.3%) | 77/243  (31.7%) | RCT | serious* | not serious | not serious | serious** | none | RR 1. 24  (0.97-1.58) | ⨁⨁◯◯  Low | CRITICAL |

*CI: Confidence interval; RR: Risk ratio; CCR Conventional Care Regimens*

**Inadequate allocation concealment and blinding in some trials*

*** Confidence intervals that includes appreciable benefits and harms*

**Table S3: Azacitidine compared to decitabine for elderly patients diagnosed with AML**

|  | **No. of patients** | | **Certainty assessment** | | | | | | **Effect** | **Certainty** | **Importance** |
| --- | --- | --- | --- | --- | --- | --- | --- | --- | --- | --- | --- |
| **No of studies** | **azacitidine** | **decitabine** | **Study**  **design** | **Risk of bias** | **Inconsistency** | **Indirectness** | **Imprecision** | **Other**  **considerations** | **Relative**  **(95% CI)** |  |  |
| Death | | | | | | | | | | | |
| 3 | 223/296  (75.3%) | 219/242  (90.5%) | RCT | serious* | not serious | not serious | serious** | none | RR 0.83  (0.77-0.90) | ⨁⨁◯◯  Low | CRITICAL |
| Complete response | | | | | | | | | | | |
| 3 | 77/296  (26.0%) | 38/242  (15.7%) | RCT | serious* | not serious | not serious | serious** | none | RR 1.66  (1.17-2.35) | ⨁⨁◯◯  Low | CRITICAL |
| Partial response | | | | | | | | | | | |
| 2 | 3/296  (1.2%) | 6/242  (2.4%) | RCT | serious* | not serious | not serious | serious** | none | RR 0.50  (0.13-1.98) | ⨁⨁◯◯  Low | CRITICAL |
| Anemia | | | | | | | | | | | |
| 3 | 67/296  (22.6%) | 79/242  (33.1%) | RCT | serious* | not serious | not serious | serious** | none | RR 0.68  (0.52-0.90) | ⨁⨁◯◯  Low | CRITICAL |
| Febrile Neutropenia | | | | | | | | | | | |
| 2 | 66/296  (27.3%) | 57/242  (23.6%) | RCT | serious* | not serious | not serious | serious** | none | RR 1.16  (0.86-1.58) | ⨁⨁◯◯  Low | CRITICAL |
| Neutropenia | | | | | | | | | | | |
| 3 | 95/296  (32.1%) | 76/242  (31.4%) | RCT | serious* | not serious | not serious | serious** | none | RR 1.02  (0.80-1.31) | ⨁⨁◯◯  Low | CRITICAL |
| Thrombocytopenia | | | | | | | | | | | |
| 3 | 104/296  (35.1%) | 95/242  (39.3%) | RCT | serious* | not serious | not serious | serious** | none | RR 0. 90  (0.72-1.12) | ⨁⨁◯◯  Low | CRITICAL |

*CI: Confidence interval; RR: Risk ratio; CCR Conventional Care Regimens*

**Inadequate allocation concealment and blinding in some trials*

*** Confidence intervals that includes appreciable benefits and harms*

**Table S4: Search Strategies.**

| Data source | Search terms |
| --- | --- |
| PubMed(n=56) | #1 "Azacitidine"[Mesh]  #2 azacitidine[Title/Abstract]  #3 ("Azacitidine"[Mesh]) OR azacitidine[Title/Abstract]  #4 "Decitabine"[Mesh]  #5 Decitabine[Title/Abstract]  #6 ("Decitabine"[Mesh]) OR Decitabine[Title/Abstract]  #7 #3 OR #6  #8 "Leukemia, Myeloid, Acute"[Mesh]  #9 acute myeloid leukemia[Title/Abstract] OR AML[Title/Abstract]  #10 ("Leukemia,Myeloid,Acute"[Mesh]) AND (acute myeloid leukemia  [Title/Abstract] OR AML[Title/Abstract])  #11 random*[Title/Abstract]  #12 "Randomized Controlled Trial"[Publication Type]  #13 #11 OR #12  #14 #7 AND #10 AND #13 |
| Embase (n=546) | #1 'azacitidine'/exp  #2 'azacitidine':ab,ti  #3 #1 OR #2  #4 'decitabine'/exp  #5 'decitabine':ab,ti  #6 #4 OR #5  #7 #3 OR #6  #8 'acute myeloid leukemia'/exp  #9 'acute myeloid leukemia':ab,ti OR 'AML':ab,ti  #10 #8 OR #9  #11 'random*':ab,ti  #12 'randomized controlled trial'/exp  #13 #11 OR #12  #14 #7 AND #10 AND #13 |
| Medline (n=67) | #1 su(Azacytidin*) OR su(Azacitidin*) OR su(ladakamycin) OR  su(mylosar) OR su(u 18496) OR su(u18496) OR su(Vidaza) OR  su(NSC-102816) OR su(NSC 102816) OR su(NSC102816)  #2 su(Deoxycytidine) OR su( 5-AzadC) OR su( AzadC Compound) OR  su( 5AzadC) OR su( 2'-Deoxy-5-azacytidine) OR su( 2' Deoxy 5  azacytidine) OR su( 5-Azadeoxycytidine) OR su( 5 Azadeoxycytidine)  OR su( Dacogen) OR su( 5-Deoxyazacytidine) OR su( 5  Deoxyazacytidine) OR su( NSC 127716) OR su( NSC-127716) OR  su( NSC127716) OR su( Decitabine Mesylate)  #3 #1 OR #2  #4 su(acute granulocytic leukaemia) OR su( acute granulocytic  leukemia) OR su( acute myeloblastic leukaemia) OR su( acute  myeloblastic leukemia) OR su( acute myelocytic leukaemia) OR  su( acute myelocytic leukemia) OR su( acute myelogenous  leukaemia) OR su( acute myelogenous leukemia) OR su( acute  myeloid leukaemia) OR su( acute nonlymphoblastic leukaemia) OR  su( acute nonlymphoblastic leukemia) OR su( acute nonlymphocytic  leukaemia) OR su( acute nonlymphocytic leukemia)  #5 (randomised controlled study) OR (randomised controlled trial) OR  (randomized controlled study) OR RCT OR random*  #6 #3 AND #4 AND #5 |
| Web of science  (n=161) | #1 TS=(Azacytidin*) OR TS=(Azacitidin*) OR TS=(ladakamycin) OR  TS=(mylosar) OR TS=(u 18496) OR TS=(u18496) OR TS=(Vidaza) OR  TS=(NSC-102816) OR TS=(NSC 102816) OR TS=(NSC102816)  #2 TS=(Deoxycytidine) OR TS=( 5-AzadC) OR TS=( AzadC Compound)  OR TS=( 5AzadC) OR TS=( 2'-Deoxy-5-azacytidine) OR TS=( 2'  Deoxy 5 azacytidine) OR TS=( 5-Azadeoxycytidine) OR TS=( 5  Azadeoxycytidine) OR TS=( Dacogen) OR TS=( 5-Deoxyazacytidine)  OR TS=( 5 Deoxyazacytidine) OR TS=( NSC 127716) OR TS=( NSC-  127716) OR TS=( NSC127716) OR TS=( Decitabine Mesylate)  #3 #1 OR #2  #4 TS=(acute granulocytic leukaemia) OR TS=(acute granulocytic  leukemia) OR TS=(acute myeloblastic leukaemia) OR TS=(acute  myeloblastic leukemia) OR TS=(acute myelocytic leukaemia) OR  TS=(acute myelocytic leukemia) OR TS=(acute myelogenous  leukaemia) OR TS=(acute myelogenous leukemia) OR TS=(acute  myeloid leukaemia) OR TS=(acute nonlymphoblastic leukaemia) OR  TS=(acute nonlymphoblastic leukemia) OR TS=(acute  nonlymphocytic leukaemia) OR TS=(acute nonlymphocytic leukemia)  #5 ALL=(randomised controlled study) OR ALL=(randomised controlled  trial) OR ALL=(randomized controlled study) OR ALL=(random*) OR  ALL=(RCT)  #6 #3 AND #4 AND #5 |
| Cochrane library  (n=131) | #1 MeSH descriptor: [Azacitidine] explode all trees  #2 (Azacitidine):ti,ab,kw  #3 #1 OR #2  #4 MeSH descriptor: [Decitabine] explode all trees  #5 ("decitabine"):ti,ab,kw  #6 #4 OR #5  #7 MeSH descriptor: [Leukemia, Myeloid, Acute] explode all trees  #8 ("acute myeloid leukemia"):ti,ab,kw  #9 #7 OR #8  #10 MeSH descriptor: [Randomized Controlled Trial] explode all trees  #11 ("random*"):ti,ab,kw  #12 #6 AND #9 AND #11 |
